# Identification of 76 novel B1 metallo-β-lactamases through large-scale screening of genomic and metagenomic data

**DOI:** 10.1186/s40168-017-0353-8

**Published:** 2017-10-12

**Authors:** Fanny Berglund, Nachiket P. Marathe, Tobias Österlund, Johan Bengtsson-Palme, Stathis Kotsakis, Carl-Fredrik Flach, D G Joakim Larsson, Erik Kristiansson

**Affiliations:** 1 Department of Mathematical Sciences, Chalmers University of Technology, Gothenburg, Sweden; 2 Centre for Antibiotic Resistance Research (CARe), University of Gothenburg, Gothenburg, Sweden; 3 Department of Infectious Diseases, Institute of Biomedicine, the Sahlgrenska Academy, University of Gothenburg, Gothenburg, Sweden

**Keywords:** β-lactam resistance, Carbapenemases, Shotgun metagenomics, Resistome, Hidden Markov model

## Abstract

**Background:**

Metallo-β-lactamases are bacterial enzymes that provide resistance to carbapenems, the most potent class of antibiotics. These enzymes are commonly encoded on mobile genetic elements, which, together with their broad substrate spectrum and lack of clinically useful inhibitors, make them a particularly problematic class of antibiotic resistance determinants. We hypothesized that there is a large and unexplored reservoir of unknown metallo-β-lactamases, some of which may spread to pathogens, thereby threatening public health. The aim of this study was to identify novel metallo-β-lactamases of class B1, the most clinically important subclass of these enzymes.

**Results:**

Based on a new computational method using an optimized hidden Markov model, we analyzed over 10,000 bacterial genomes and plasmids together with more than 5 terabases of metagenomic data to identify novel metallo-β-lactamase genes. In total, 76 novel genes were predicted, forming 59 previously undescribed metallo-β-lactamase gene families. The ability to hydrolyze imipenem in an *Escherichia coli* host was experimentally confirmed for 18 of the 21 tested genes. Two of the novel B1 metallo-β-lactamase genes contained atypical zinc-binding motifs in their active sites, which were previously undescribed for metallo-β-lactamases. Phylogenetic analysis showed that B1 metallo-β-lactamases could be divided into five major groups based on their evolutionary origin. Our results also show that, except for one, all of the previously characterized mobile B1 β-lactamases are likely to have originated from chromosomal genes present in *Shewanella* spp. and other Proteobacterial species.

**Conclusions:**

This study more than doubles the number of known B1 metallo-β-lactamases. The findings have further elucidated the diversity and evolutionary history of this important class of antibiotic resistance genes and prepare us for some of the challenges that may be faced in clinics in the future.

**Electronic supplementary material:**

The online version of this article (10.1186/s40168-017-0353-8) contains supplementary material, which is available to authorized users.

## Background

Carbapenems are broad-spectrum β-lactams that are often used when treating life-threatening infections or as a last resort for infections caused by multidrug resistant bacteria [1, 2]. Resistance to carbapenems is mediated by carbapenemases, which are β-lactamases that hydrolyze the β-lactam ring of carbapenem antibiotics [3]. Carbapenemases are present in three of the four molecular classes of β-lactamases, and while carbapenemases in classes A and D use a catalytic serine for hydrolysis, the class B enzymes (metallo-β-lactamases), which all are carbapenemases, are dependent on zinc ions for their activity [4]. Metallo-β-lactamases have broad-spectrum activity profiles, and the majority of these enzymes are able to hydrolyze penicillins and cephalosporins in addition to carbapenems. Furthermore, there are no clinically available inhibitors that can block the metallo-β-lactamase activity, which makes the class B metallo-β-lactamases a particularly worrisome class of resistance genes [5].

Metallo-β-lactamases can be phylogenetically divided into three subclasses, B1, B2, and B3 [6]. Metallo-β-lactamases from subclass B1 (B1BL) and subclass B3 use two zinc ions within their active sites and have a much broader resistance spectrum compared to subclass B2, which uses only one zinc ion [3]. Furthermore, the vast majority of the mobile class B carbapenemases identified to date belong to subclass B1, including the clinically important IMP (for “active on imipenem”) and VIM (Verona integron-encoded metallo-β-lactamase) gene families that are commonly found in integrons, transposons, and plasmids [7, 8]. The more recently identified B1BL gene family NDM (New Dehli metallo-β-lactamase), first discovered in 2009 on a plasmid in a *Klebsiella pneumoniae* strain [9], has spread globally in the span of only a few years and today is found in multidrug-resistant bacteria in many countries [10], underscoring the increasing clinical importance of surveillance of carbapenemases from the B1 subclass.

Environmental and commensal bacterial communities are known to maintain a large diversity of clinically relevant antibiotic resistance genes [11, 12]. This diversity is known to be especially large in environments with an antibiotic selection pressure, such as environments polluted with antibiotics from the production of pharmaceuticals and wastewater treatment plants [13–15]. In addition to the already known resistance genes, bacterial communities also harbor a wide range of novel resistance determinants that have yet to be encountered in clinical settings [16–18]. If mobilized, these genes may be transferred to pathogens, either directly or indirectly via commensal bacteria in humans or animals, which can lead to infections that are difficult or impossible to treat [2]. Indeed, previously uncharacterized β-lactamases, including class B carbapenemases, have been found in bacterial communities sampled from Alaskan, apple orchard, and agricultural soils and cow manure [19–22]. It is therefore likely that current knowledge regarding B1BLs only reflects “the tip of the iceberg” and that the full diversity of these enzymes is far from being completely described. This is further emphasized by the fact that many original hosts of the currently known mobile B1BL genes have not yet been identified, making their evolutionary origins unclear. Further examination of environmental and commensal bacteria in search of potentially new B1BLs is therefore important and would enable the identification and surveillance of potent genes before they are mobilized and horizontally transferred into pathogens. Expanding the number of known chromosomal and mobile B1BL genes would also provide a more detailed phylogenetic view of this gene class and facilitate the further elucidation of their origin and evolutionary history [23].

The amount of genomic data present in public repositories has rapidly accumulated in recent years and for bacteria alone, the amount of data deposited in GenBank has shown a yearly increase of approximately 50% [24]. The same is true for metagenomic data available in public repositories, which has grown in size due to recent breakthroughs in high-throughput DNA sequencing [25]. In contrast to sequencing of individual strains, metagenomics enables the characterization of entire communities, including the large proportion of bacteria that are hard to cultivate under normal laboratory conditions [26]. However, the fragmented nature of metagenomic data makes it a non-trivial task to search for novel genes. This is especially true for B1BL genes, for which the sequence similarity between gene families can be as low as 28% [27]. Thus, a large proportion of the sequence data in the public repositories has not been analyzed for novel B1BLs, despite the fact these data are likely to contain a large number of previously undescribed gene variants.

In this study, we aim to substantially extend our knowledge of B1BLs by searching for previously undescribed genes in available bacterial DNA sequence data. To enable the analysis of sequences from both isolated strains as well as fragmented metagenomic data, we developed a sensitive computational method that can identify conserved evolutionary patterns from genes within the B1BL subclass using a hidden Markov model (HMM). Cross-validation was used to optimize the parameters of the model to identify previously undescribed B1BL genes with high accuracy. The method was then used to search bacterial genomes and plasmids present in the NCBI GenBank RefSeq database as well as more than five terabases of metagenomic data from human and environmental bacterial communities. In total, we identified 76 novel B1BL genes that could be divided into 59 novel gene families. Twenty-one of the genes were selected for experimental validation, 86% (18 of 21) of which were confirmed to yield imipenem-hydrolyzing activity when expressed in an *Escherichia coli* host. Two of the novel genes were located within a genetic context that suggests they may be mobile. Phylogenetic analysis of the combined set of previously described and novel metallo-β-lactamases showed that the B1BLs can be organized into five distinct groups that are largely defined by the taxonomy of their hosts. The results of this study substantially increase the number of identified B1BLs, some of which may pose a threat to public health in the future and provides a more detailed picture of their diversity and evolutionary history.

## Results

In this study, we applied a computational method to predict novel B1BLs in genomes and metagenomes. The method was designed based on a sensitive hidden Markov model (HMM) created from previously known B1BL genes with verified activity against carbapenems (see “Methods” section, Additional file 1: Figure S1). The method is applicable to both genomes and metagenomes, and the output consists of predicted full-length B1BL genes. Cross-validation was used to optimize the true positive rate to identify previously undescribed B1BL genes. For full-length genes, the method correctly classified all tested B1BL genes (an estimated true positive rate of 100%), while the false positive rate (FPR) was very low, even for closely related genes from the MBL superfamily [23] (an FPR of 0% for all tested gene groups within the MBL superfamily). For metagenomic data, using a fragment length of 100 nucleotides, the method showed a true positive rate of 89% and a FPR of 4% and 0% for the MBL superfamily and random genomic fragments, respectively.

The method was used to search for novel B1BL genes in bacterial genomes, plasmids, and other bacterial DNA sequences present in the NCBI GenBank repository. From sequences that were present in NCBI RefSeq bacteria, consisting of 2744 complete genomes and 162 draft genomes, 92 B1BL genes were predicted (Table 1). In total, 88 genomes (3.2%) and 2 plasmids carried one B1BL gene, and one genome carried two B1BL genes (*Shewanella denitrificans* strain OS217). The occurrence of B1BL genes was significantly higher in Gram-negative bacteria (5.3%) compared to Gram-positive bacteria (1.7%) (*p* = 0.0022, Fisher’s exact test). In addition, there was a strong overrepresentation of B1BL-carrying bacteria in the phylum Bacteroidetes (22.4%) compared to bacteria from other phyla (3.0%) (*p* = 1.17 × 10^−9^, Fisher’s exact test). When Bacteroidetes was excluded from the Gram-negative set, there was still a tendency towards an overrepresentation of B1BL-carrying Gram-negative bacteria (*p* = 0.0713, Fisher’s exact test). We found, however, no association between the distribution of B1BL genes and human-associated bacteria or pathogens as defined by the PATRIC database [28] (*p* = 0.8695 and *p* = 0.6021, respectively).

**Table 1:** Summary of the analyzed genome datasets and predicted B1BL genes

Data set	Size (nt)	Sequences	Predicted B1BL genes	Families^a^		Description
				Known	New	
Bacteria	9.23 × 10^9^	5.24 × 10^3^	92	7	35	NCBI RefSeq bacteria [73]
Plasmid	5.22 × 10^8^	9.23 × 10^3^	148	7	1	NCBI RefSeq plasmid [73]
NT	5.22 × 10^10^	2.07 × 10^7^	944	21	36	NCBI NT [74]
EnvNT	9.55 × 10^9^	2.07 × 10^7^	15	1	13	NCBI environmental NT [74]
Total	7.19 × 10^10^	4.15 × 10^7^	266^b^	21^b^	50^b^	

^a^Based on a sequence similarity cut-off of  70%

^b^Non-redundant genes

Among the predicted B1BL genes in RefSeq bacteria, one was located on the chromosome of *Pseudomonas stutzeri* DSM 10701. This gene was found to be most closely related to the previously identified mobile B1BL gene KHM-1 (54% amino acid sequence identity). Interestingly, this gene was located on a genomic insert that was present in only one out of six *P*. *stutzeri* genomes available in NCBI GenBank. In addition to the B1BL gene, the insert also contained a gene encoding the protein domain NERD, associated with endonucleases previously found on virulence plasmids [29], as well as genes belonging to the Fic/DOC domain family (pfam02661), previously connected with a toxin-antitoxin module from the *E*. *coli* prophage P1 [30].

Among the NCBI RefSeq plasmids, consisting of 9225 sequences, we predicted 148 B1BL genes, of which 147 were associated with previously known gene families (Table 1). One identified gene, located on a plasmid from *Myroides odoratimimus* strain PR63039, had a sequence identity lower than 70% to any previously described B1BL. This plasmid also harbored the gene *tet(*X), which confers resistance to tetracycline and tigecycline, another last-resort broad-spectrum antibiotic [31, 32] and genes associated with class B type IV secretion systems (TraM_B_) [33]. The analysis of the NT and EnvNT GenBank databases resulted in the identification of 959 additional predicted B1BL genes, bringing the total number predicted B1BL genes to 1199, corresponding to 266 non-redundant sequences (Table 1). Clustering using a sequence similarity cut-off of 70% showed that these genes formed 71 families, 50 of which were previously uncharacterized.

**Fig. 1:**
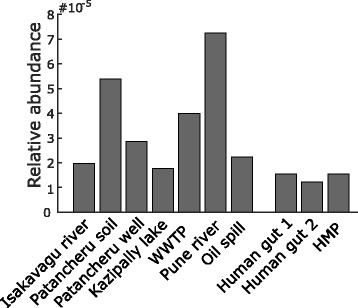
The relative abundance of B1BL gene fragments in the analyzed metagenomic data. The relative abundance of B1BL gene fragments varied between 13.8 and 79.0 per million metagenomic fragments. There was a significant difference in abundance between the environmental metagenomes (left) and the human microbiome (right) (*p* = 0.0167, Wilcoxon rank sum test). The highest levels were observed in the river sediments sampled close to the effluent of a hospital in Pune, India (“Pune river”)

Next, the search method was used to identify novel B1BL genes in 5.7 terabases of short sequence reads from metagenomes (Table 2). The metagenomic datasets were selected to represent both the human microbiome as well as bacterial communities from different environments. Special priority was given to metagenomes from heavily polluted environments, which have previously been shown to contain a high abundance and diversity of resistance genes [13, 34, 35]. The relative abundance of B1BL gene fragments ranged from 13.8 to 79.0 B1BL fragments per million metagenomic fragments (Fig. 1). The levels were significantly higher in the environmental metagenomes compared to the human microbiome (*p* = 0.0167, Wilcoxon rank sum test). The highest abundance of B1BL gene fragments was found in the river sediment sampled close to and from Hospital effluent in Pune, India (“Pune river”). Assembly of the fragments from the metagenomic data search resulted in the identification of 16 full-length genes (Table 2). Among these genes, 13 were classified as new B1BL gene families while three were previously characterized (NDM-1, IMP-37, and EBR-1).

**Table 2:** Summary of the analyzed metagenomes and predicted B1BL genes

Data set	Size (nt)	Reads	Predicted B1BL genes	Families^a^		Description
				Known	New	
Isakavagu river	3.89 × 10^10^	3.85 × 10^8^	0	0	0	Polluted river sediment [15]
Kazipally lake	6.75 × 10^9^	6.68 × 10^7^	0	0	0	Polluted lake [13]
Patancheru soil	4.73 × 10^10^	4.68 × 10^8^	0	0	0	Soil [62]
Patancheru well	7.32 × 10^10^	7.25 × 10^8^	3	0	3	Well water [62]
WWTP	4.82 × 10^11^	5.18 × 10^9^	8	2	6	Sewage treatment plants [14]
Oil spill	2.75 × 10^11^	2.72 × 10^9^	1	0	1	Oil spill [61]
Pune river	3.91 × 10^11^	3.11 × 10^9^	4	1	3	River water [63]
Human gut 1	3.71 × 10^11^	5.22 × 10^9^	0	0	0	Human gut [59]
Human gut 2	2.74 × 10^11^	3.41 × 10^9^	0	0	0	Human gut [60]
HMP	4.69 × 10^12^	4.41 × 10^10^	0	0	0	Human microbiome [53]
Total	5.65 × 10^12^	6.54 × 10^10^	16	3	13	

^a^Based on a sequence similarity cut-off of  70%

In total, the analysis of the genomic and metagenomic data resulted in the identification of 279 unique B1BL genes, of which 76 were novel. Of these novel genes, 21 were selected for experimental verification (Table 3). Each gene was synthesized, transformed into an *E*. *coli* host, and then the imipenem hydrolyzing activity was assessed using the Carba NP test. In total, 18 of the 21 selected genes (86%) were able to hydrolyze imipenem, while three genes (G24, G65, and G69) did not show any enzymatic activity (Table 3). In addition, the *P*. *stutzeri* DSM 10701 strain, which was predicted to contain a novel B1BL gene, yielded a positive Carba NP test.

**Table 3:** Summary of the 22 experimentally verified B1BL genes

Source data set	Gene ID	Predicted family	Group	Proposed name	Protein length (aa)	Positive Carba NP test
RefSeq plasmid	G04	4	B1–3	MYO-1	266	Yes
RefSeq bacteria	G06	6	B1–2	SHD-1	265	Yes
RefSeq bacteria	G09	9	B1–5	SPS-1	263	Yes
RefSeq bacteria	G12	11	B1–1	MYX-1	262	Yes
RefSeq bacteria	G13	12	B1–1	STA-1	262	Yes
RefSeq bacteria	G24	17	B1–3		259	No
RefSeq bacteria	G27	20	B1–1	ANA-1	258	Yes
RefSeq bacteria	G28	21	B1–3	ECV-1	258	Yes
RefSeq bacteria	G29	22	B1–3	ORR-1	256	Yes
RefSeq bacteria	G31	24	B1–4	FIA-1	254	Yes
WWTP	G33	25	B1–3		252	Yes
WWTP	G37	29	B1–4		251	Yes
RefSeq bacteria	G52	36	B1–3	ZOG-1	247	Yes
Patancheru well	G58	41	B1–4		245	Yes
RefSeq bacteria	G63	46	B1–2	TTU-1	244	Yes
Pune river	G65	48	B1–3		242	No
RefSeq bacteria	G67	50	B1–2	PST-1	243	Yes^a^
RefSeq bacteria	G69	52	B1–3		242	No
Patancheru well	G70	53	B1–2		242	Yes
Oil spill	G71	54	B1–2		240	Yes
RefSeq bacteria	G74	57	B1–2	SHN-1	238	Yes
RefSeq bacteria	G77	ALI	B1–2	ALI-2	246	Yes

^a^Strain tested

**Fig. 2:**
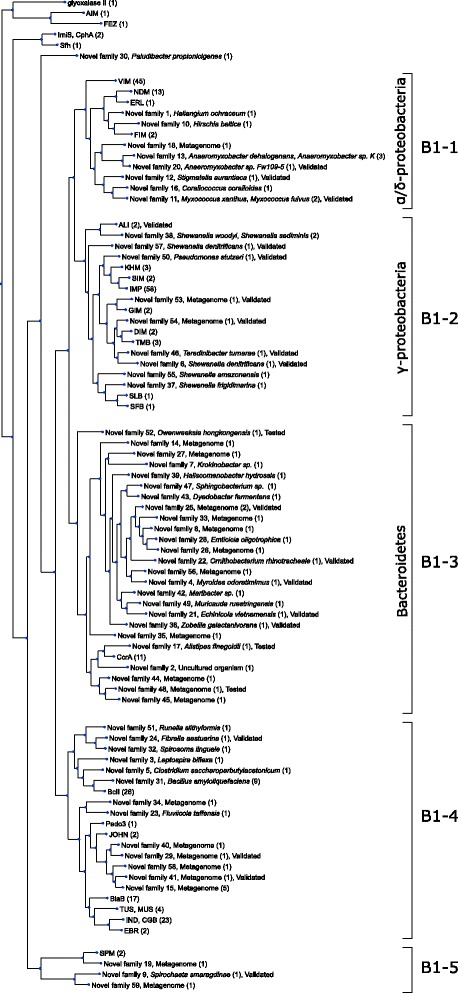
A phylogenetic tree describing the evolutionary relationship between the B1 metallo-β-lactamases predicted in this study. The tree was created from representative sequences for the gene families generated by clustering of predicted and previously characterized B1BL at a 70% amino sequence identity cutoff (see “Methods” section). Previously characterized B1BL genes are annotated with the gene name. Novel gene families that were predicted in this study are annotated with the corresponding family number and the sources (metagenome or species name of the host). The numbers in parenthesis indicate how many unique genes there are in each family. The tree was divided into 5 groups (B1–1 to B1–5) as referred to in the text. Complete information on the predicted genes and families are available in Additional file 2: Table S1

The 76 predicted and previously uncharacterized B1BL genes clustered into 59 novel families (Additional file 2: Table S1), resulting in a total of 81 B1BL families described to date. The protein lengths of the families ranged from 236 aa (G58 and EBR-1) to 278 aa (G01), with an average of 251 aa. The sequence identity between the families, old and new, ranged from 20.35 up to 66.67%. After combining the previously characterized B1BL gene families with a representative sequence from each novel family predicted in this study, phylogenetic analysis revealed five larger groups, denoted here as B1–1 to B1–5 (Fig. 2).

The first two groups, B1–1 and B1–2, contained 17 novel B1BL families together with many of the mobile B1BLs, including NDM, FIM, VIM, KHM, IMP, SIM, GIM, DIM, and TMB [3, 36]. The previously characterized, chromosomally encoded B1BL families ALI, SLB, SFB, and ERL were also present in these groups. All identified bacterial hosts of the chromosomal genes in these two groups belonged to the phylum Proteobacteria. While the hosts of the B1–1 group all belonged to the Alpha- and Deltaproteobacteria, the identified hosts of the B1–2 group all belonged to the taxonomic class Gammaproteobacteria, with the genus *Shewanella* accounting for 67% of the genes.

The third group (B1–3) consisted of 25 novel B1BL families and one previously characterized B1BL family (CcrA). All bacterial hosts of the chromosomal genes in this group, both the representative genes and the members of the families, belonged to the phylum Bacteroidetes. Two of the families in this group consisted of genes located in pathogens, where G29 in family 22 was found on the chromosome of an isolate from the poultry pathogen *Ornithobacterium rhinotracheale* [37] and G04 in family 4 was present on a plasmid harbored by an isolate of the opportunistic pathogen *Myroides odoratimimus* [38].

The B1–4 group was formed from 13 novel families and seven previously known chromosomal genes, including BcII, EBR, IND, TUS, BlaB, JOHN, and PEDO3 [21, 39]. Among the 13 novel families, six were identified from environmental sequence data (EnvNT and environmental metagenomes). The hosts carrying chromosomal genes were from the Bacteroidetes and Firmicutes phyla.

The fifth group (B1–5) was the smallest and consisted of only four B1BL families, of which three were novel and one was known (the plasmid-mediated SPM-1). Interestingly, two of the predicted genes (G09 in family 9 and G76 in family 59) had an atypical zinc-binding motif in which the residue H116, present in previously reported B1BL genes [27], was replaced with a glycine. One of these genes, G09, was confirmed to have imipenem-hydrolyzing activity when expressed in *E*. *coli*. These two genes were the only sequences in this analysis where the zinc-binding motif differed.

## Discussion

This study substantially extends our knowledge of the diversity within the B1 subclass of metallo-β-lactamases. By exploring 2906 bacterial genomes, 9230 plasmid sequences and more than 5 terabases of metagenomic data, we predicted 76 previously uncharacterized B1BL genes with less than 70% amino acid identity to previously known metallo-β-lactamases. These genes were divided into 59 novel families, revealing that the diversity within the B1BL subclass of metallo-β-lactamases is far greater than what has previously been described. Experimental validation of 21 selected genes showed that 18 (86%) were able to hydrolyze imipenem when expressed in an *E*. *coli* host. This provides strong confidence to the applied computational method. In addition, the predicted enzymatic activity was also confirmed for the gene present in the strain *P*. *stutzeri* DSM 10701. The phylogenetic analysis revealed that all but one (G38, family 30) of the novel B1BL genes identified in this study, together with the previously known B1BL genes, could be divided into five main groups, which could be largely explained by the taxonomic affiliation of their corresponding hosts. Proteobacteria, which were represented by two large groups in the phylogenetic tree, dominated, followed by Bacteroidetes. All mobile B1BL families, except for SPM, were located in the two Proteobacteria-associated groups, indicating that the clinically important mobile B1BL genes originated in Proteobacteria and that, in the future, the host range for mobile B1BL genes acquired by pathogens may be limited. Interestingly, Bacteroidetes was found to be highly overrepresented in relation to the number of genomes present in the databases, suggesting that they more frequently carry B1BLs. Furthermore, we also found that B1BLs were significantly overrepresented among Gram-negative bacteria compared to Gram-positive. However, we did not find any overrepresentation of B1BL in pathogens or human-associated bacteria. These results substantially increase the number of gene families of subclass B1 [39] and provides a more detailed view of the evolutionary history of this important subclass of metallo-β-lactamases.

The genetic contexts of two of the novel B1BL genes suggest that they may be mobile. First, the B1BL gene G67 (family 50) (in this study named PST-1), found on the chromosome of *P*. *stutzeri* DSM 10701, phylogenetically clustered with the previously characterized mobile B1BL families KHM, SIM, and IMP. The gene was located on a structurally variable region that was absent in the five other *P*. *stutzeri* strains present in the RefSeq database. In addition, no homology could be found with other species within the *Pseudomonas* genus, and no known integrons or transposons could be detected in the up- or downstream regions of the gene. There was, however, weak homology to two other genes located on the same insert as the predicted β-lactamase. These genes, NERD and Fic, have previously been found on plasmids, phages, and associated with integrons and have been suggested to code for mechanisms related to the horizontal transfer of genetic material [30, 40]. *Pseudomonas* is notorious for carrying mobile class B β-lactamases [41, 42], and several of the known mobile B1BL families were initially identified in this genus (IMP, VIM, SPM, and GIM) [43]*.* Thus, our results indicate that the production of PST-1 by *P*. *stutzeri* DSM 10701 may be the result of a recent gene acquisition event and that this gene could therefore, potentially, be a member of a previously uncharacterized mobile B1BL family.

Second, the gene G04 (in this study named MYO-1) in family 4 was located, together with a tetracycline resistance gene, on a plasmid in the genome of the opportunistic pathogen *M*. *odoratimimus* strain PR63039. This gene phylogenetically belonged to group B1–3, along with the chromosomal Bacteroidetes BLB1 genes, and was the only mobile gene in this cluster. Furthermore, the plasmid also contained parts of a class B conjugation system, which is known to be predominantly found in the Bacteroidetes phylum [33]. Thus, our results suggest that this gene was potentially mobilized from a species within the Bacteroidetes phylum and that its host-range may be limited at present. However, when validated in *E*. *coli*, the gene showed enzymatic activity despite no codon optimization, demonstrating that it is functional in a Proteobacterial host. Gene transfer events between Bacteroidetes and Gram-positive bacteria have been previously suggested to occur [44], and therefore, it cannot be excluded that this gene may be, or become, transferable to pathogens outside Bacteroidetes. This gene may thus constitute an important factor to be considered for clinically relevant carbapenem resistance in the future. The remaining B1BL genes predicted in this study did not have any clear indications regarding their mobility with respect to their genetic context or their location in the phylogenetic tree. However, the majority of the tested B1BLs exhibited enzymatic activity in *E*. *coli*. Thus, if these genes were to become mobilized and spread to pathogenic bacteria, they may constitute a threat to human health in the future [45]. Furthermore, the large increase in known chromosomal B1BL genes will enable more detailed phylogenetic studies of the B1 subclass of metallo-β-lactamases [23], an essential step to further elucidate the evolution and origins of mobile B1BL genes.

The phylogenetic analysis showed that 9 out of the 10 known mobile B1BL gene families clustered together with chromosomal B1BL genes in Proteobacteria (groups B1–1 and B1–2; the only exception was SPM-1). Interestingly, the B1–2 group, which contained 6 of the known mobile B1BLs, was highly overrepresented by chromosomal genes from the *Shewanella* genus. *Shewanella* has previously been hypothesized to be the origin of mobile antibiotic resistance genes, including the class D carbapenemase OXA-48 (*Shewanella oneidensis*, [46]) and the fluoroquinolone resistance gene *qnrA* (*Shewanella algae* [47]). Thus, our results suggest that many of the known mobile B1BL genes likely originated from hosts associated with the phylum Proteobacteria, especially *Shewanella* spp. In addition, our results suggest that clinically relevant mobile B1BL genes originating from hosts outside Proteobacteria are rare. This could potentially be due to the many barriers preventing genes from being efficiently transferred between phyla. The beta-lactamase protein needs to be transported outside the cytoplasmic membrane of the cell to induce a resistance phenotype, and its signal peptides therefore need to be recognized by the host [48, 49]. β-Lactamases also need to be highly expressed to provide high levels of resistance, which requires a codon distribution that is suitable for the host cell [50]. In fact, all of the predicted genes that showed negative Carba NP tests were located in the Bacteroidetes group B1–3, and two of them contained codons that are rare in *E*. *coli*. Since the experimental validation was based on the identified protein sequences and no codon optimization was applied, rare codons could potentially explain the lack of observed enzymatic activity. Another explanation could be that their signal peptides were not recognized by *E*. *coli*. Thus, it is plausible that many B1BL genes from distant phyla may be less suitable for Proteobacterial hosts and may therefore not yield any substantial resistance phenotype. Furthermore, many forms of conjugative elements, which are the primary mechanisms for the transfer of antibiotic resistance genes between cells, have a limited host range that can prevent sharing of genes between phylogenetically distant species [51, 52]. Indeed, the identified bacterial hosts carrying the mobile B1BLs IMP and VIM all belong to the phylum Proteobacteria. Nevertheless, our validation showed that 6 of 8 (75%) of the chromosomally located genes from phyla other than Proteobacteria were able to hydrolyze imipenem in an *E*. *coli* host, suggesting that no further evolution is needed for them to be functional should they be horizontally transferred. This suggests that, at least in theory, the recruitment of B1BL genes outside of Proteobacteria should be possible. It should, however, be pointed out that such genes may pose a significant risk if present in, or transferred to, pathogens belonging to their original phylum. Furthermore, we noted that none of the known mobile B1BL genes had a close match to a chromosomal gene in a known pathogenic or non-pathogenic bacterial species. This suggests that the known mobile B1BL genes either were not recently mobilized or originated from species with yet uncharacterized genomes. Given that a large proportion of the pathogenic- and human-associated bacteria have been sequenced to date [53], it is plausible that many of the mobile B1BL genes have an environmental origin. Nevertheless, our results underscore the fact that Proteobacteria, in particular *Shewanella*, constitutes an important reservoir for the mobilization of B1BLs.

In B1 enzymes, the Zn(II) ion at the first binding site is coordinated to the residues His116, His118, and His196, according to the standard BBL numbering scheme [27], while the second binding site for the second Zn(II) ion is coordinated by the residues Asp120, Cys221, and His263. Interestingly, two of the novel B1BL genes, G09 (here named SPS-1, in family 9) and G76 (in family 59), which were both located in the fifth group (B1–5), had the conserved His116 replaced by a glycine, which has previously not been reported in any class B β-lactamase. One of these enzymes was selected for validation (SPS-1 in family 9; see Table 3 and Additional file 2: Table S1), and its imipenem hydrolyzing activity was confirmed when expressed in *E*. *coli*. Metallo-β-lactamases in the closely related B2 subclass are known to have the His116 replaced by asparagine [3]. Mutational analysis of the subclass B2 gene CphA has shown that the replacement of asparagine with histidine at position His116 resulted in a 12- and 260-fold increase in activity against penicillins and cephalosporins, respectively [54]. Thus, it cannot be excluded that the changes in the zinc-binding sites of SPS-1 and G76, and potentially other yet to be described β-lactamases within this group, may have significantly different enzymatic activities and substrate spectra. It should be noted that, based on the current paradigm of three separate B subclasses (B1, B2, and B3), our study clearly classified SPS-1 and G76 as B1 β-lactamases. Thus, our results suggest that the B1 subclass has a higher diversity of zinc-binding sites than what has previously been described in the literature.

Thirteen of the predicted B1BL genes were identified from metagenomic data, all of which were assembled from environmental samples, primarily polluted ones. The abundance of B1BL gene fragments was also found to be three-fold higher in the environmental metagenomes compared to metagenomes from the human microbiome, although the variation between the environmental metagenomes was high. This further strengthens the hypotheses that the environment serves as an unexplored reservoir of class B β-lactamases, which is in agreement with previous studies [11, 20]. However, there were B1BL fragments found in the human microbiome, especially in stool samples, but no assembly of full-length genes was possible. To investigate if this was due to lack of sequencing depth, we attempted to pool the B1BL gene fragments from different individuals based on body site, but no significant improvement was observed. One likely reason for the inability to identify new B1BLs in the human-associated metagenomes is the combination of the low abundance of β-lactam resistance genes with the high diversity of the microbiome, both between individuals and body sites [53]. In contrast to the environmental metagenomes, where replicated samples were taken from similar, often spatially close, communities, the human-associated data used in this study were comprised of samples from a large number of individuals, each sequenced at a limited depth. Thus, deeper sequencing of the individual samples is likely necessary to identify potential variants only present in smaller proportions of individuals. However, our results indicate that many of the identified fragments are false positives and that there is likely no single widespread previously uncharacterized B1BL gene in the human microbiomes investigated.

The identification of novel β-lactamases was based on a probabilistic profile hidden Markov model (HMM) designed to identify novel B1BL gene families. Compared to more standard bioinformatics approaches to homology searches, such as BLAST, profile HMMs take the position-specific sequence conservation into account and therefore offer much higher sensitivity [55]. The model was optimized using cross-validation where the probability to identify a B1BL gene excluded from the model was maximized. This enabled us to identify novel B1BL genes, even in highly fragmented sequence data, with high accuracy. This is underscored by the experimental validation where imipenem hydrolysis was verified in 86% (18 of 21 tested genes) of the validated genes. In addition, two of the genes that showed no activity both contained several codons that are considered rare in *E*. *coli*, suggesting that they may not have been translated with sufficient efficiency and could be functional in other hosts. Taken together, it is therefore likely that the majority of the genes predicted in this study that were not subjected to validation are also novel, functional B1BLs.

## Conclusions

Computational analysis of more than 10,000 sequences from genomes, plasmids, and > 5 terabases of metagenomic data was used to identify novel B1BLs. In total, 59 novel families containing 76 novel B1BL genes were predicted, and 86% of the validated genes showed imipenem hydrolyzing activity when expressed in *E*. *coli*. Two of the predicted genes were located in genetic contexts that suggest they may be horizontally transferable. Our analysis showed that the B1BL genes can be organized into five separate phylogenetic groups and that the majority of all known mobile B1BL genes were found to be closely related to chromosomal genes of the *Shewanella* genus and other Proteobacterial species. Furthermore, two identified novel B1BL genes had an atypical zinc-binding motif in their active site that has not previously been described within class B metallo-β-lactamases. This study substantially expands the number of described B1BLs and identifies genes that, if mobilized into pathogens, could pose a threat to our ability to efficiently treat multidrug-resistant bacteria. Our results also provide a more detailed picture of the diversity and evolutionary history of B1BLs.

## Methods

### Description of the computational method

The novel B1 metallo-β-lactamase (B1BL) genes identified in this study were predicted using a computational method based on a hidden Markov model (HMM). The method was designed to take either whole genomes or metagenomes as input and provide the predicted full-length genes as an output. A flowchart summarizing the method is provided in Additional file 1: Figure S1. For complete genomes, the method searched for matches against the HMM, which were classified as B1BL depending on the score. For metagenomes, the method first performed a quality control of the reads, then searched each fragment using the HMM. Fragments classified as being derived from a B1BL gene were then assembled and elongated (if the assembled gene was not of full-length).

The HMM used to identify evolutionary conserved patterns from B1BL genes was built from a training dataset consisting of representative sequences from 20 verified genes in the B1 metallo-β-lactamase subclass (Additional file 3: Table S2). The corresponding protein sequences were downloaded from the NCBI database (http://www.ncbi.nlm.nih.gov/) during September of 2015. The training sequences were aligned with ClustalW using the default settings [56] and then were used as input (“training dataset”) for the HMM. The model was built using HMMER version 3.1b1 [57] using “hmmbuild” with the default settings.

### Optimization of the probabilistic model

The score cut-off of the model was optimized to identify novel families in genome sequences using leave-one-out cross-validation. First, a model was built from the training dataset with one gene excluded. The excluded gene was then subjected to the model and the true positive rate, defined as the proportion of correctly identified sequences at a specific score, was estimated. This process was repeated 20 times, one time for each gene in the training dataset. To estimate the specificity of the model, we assembled a “false” dataset consisting of 38 selected genes from the closest known homologs, the 17 groups of the MBL superfamily [58], which were downloaded from UniProt (http://www.uniprot.org/) and from the NCBI FTP site (ftp://ftp.ncbi.nlm.nih.gov/) during September of 2015. The false positive rate of the model was estimated based on the incorrectly classified genes. The ratio between the true and false positive rate was used to determine an optimal threshold score for a gene to be classified as B1BL.

To optimize the threshold score for the model when applied to metagenomic data, the cross-validation was additionally performed for gene fragments. The excluded B1 gene was randomly cut into 10,000 fragments, each 100 nucleotides long, corresponding to the most common read length for Illumina sequence data, and then was analyzed by the model. The true positive rate was then calculated as the proportion of correctly classified fragments. The false positive rate was estimated by both (1) fragmented genes from the MBL superfamily (as described above) and (2) fragments randomly selected from 1000 bacterial genomes (from NCBI RefSeq), from which every predicted MBL subclass B1 gene had been removed.

Based on the results of the cross-validation, the score threshold for the full-length genes was set to 135.4, corresponding to a true positive rate of 100% and a false positive rate of 0% for the MBL superfamily. For fragmented data, the score was set to 10, corresponding to a true positive rate of 89% and a false positive rate of 4 and 0% for the MBL superfamily and random genomic fragments, respectively (Additional file 4: Figure S2).

### Processing genomic and metagenomic data

Genomes and plasmids from the NCBI datasets (RefSeq Bacteria, RefSeq Plasmid, NT and Environmental NT) were downloaded from the NCBI FTP site (ftp://ftp.ncbi.nlm.nih.gov/) during the spring of 2015, except for the NCBI RefSeq plasmids, which were downloaded in the spring of 2016. The metagenomic datasets (Additional file 5: Table S3) consisted of raw Illumina sequence reads from the human gut microbiome (“Human gut 1” [59], “Human gut 2” [60]), and the human microbiome (“HMP” [53]). In addition, datasets from various polluted environments were included and consisted of oil-exposed marine sediments (“oil spill” [61]), a pharmaceutical-polluted lake (“polluted lake” [13]), Swedish waste water treatment plants (“WWTP” [14]), and pharmaceutical-polluted river sediments (“Isakavagu river” [15]). We also included samples from bacterial communities in soil and well water (“Patancheru soil,” “Patancheru well” [62]) and from river sediments in Pune (“Pune river,” [63]).

The metagenomic data were processed as follows: data in FASTQ format was quality controlled using the FASTX-toolkit version 0.13.2 [64] with “fastq_quality_filter,” in which sequences containing ambiguous bases were removed. The sequences were then converted to FASTA with “fastq_to_fasta.” After conversion, the sequences were translated into amino acid sequences in all six reading frames using EMBOSS “transeq” version 6.3.1 [65] with the parameters “-frame 6 -table 11.” All amino acid sequences were analyzed with HMMER3 using “hmmsearch” with parameters “--domtblout -E 1000 --domE 1000” using the HMM described above. The domain score, envelope start (env_start), envelope end (env_end), and sequence ID were extracted from the output of hmmsearch and were classified as a B1BL if the domain score was higher than the previously calculated score threshold.

For the metagenomic data, the fragments matching the HMM were assembled with CAP3 [66] using a strict identity cutoff of 97% and a minimal overlap of 30 nucleotides with the options “-p 97 -o 30.” If the assembled potential B1 gene was not full-length, it was extended as follows: first, the reads in the quality-filtered FASTA files were mapped to the contig using USEARCH [67] with the options “-usearch_local -strand both -id 0.7 -blast6out.” The fragments matching the contig template were collected together with their corresponding paired-end read and then assembled using SPAdes [68] with the options “--sc --only-assembler.” If the contig was too short to contain a full-length B1BL gene, the procedure was repeated up to three times.

All putative B1BL genes that were assembled from metagenomic data were subjected to an additional quality control. This was done by mapping reads from the samples from which they were identified back to the contigs with Bowtie2 [69] using the parameters “-x <templateContig.fa> -1 <retrievedReads1.fq> -2 <retrievedReads2.fq.” The physical coverage, defined as the number of spanning paired-end reads, was then calculated for each position. Contigs where the physical coverage was zero at any nucleotide position were excluded from the analysis. Finally, the full-length genes were analyzed by the model again, and genes with a score of less than the threshold for full-length genes were discarded.

### Experimental validation

To assess the functionality of the predicted novel genes, 21 of the candidate novel genes (ORFs) were synthesized at ThermoFisher Scientific, Germany, using the GeneArt gene synthesis service. The genes were subcloned into the expression vector pZE21-MCS1 [70]. The recombinant pZE21-MCS1 plasmids containing the novel resistance gene candidates were then transformed into *E*. *coli* C600Z1 (Expressys Ruelzheim, Germany) by electroporation. The expression of the cloned gene was induced using anhydroteteracycline (aTc). To assess imipenem hydrolysis, Carba NP tests were carried out on overnight cultures grown on Mueller Hilton agar plates containing 100 ng/ml of aTc [71]. Imipenem was procured from Glentham Life Sciences, UK. An *E*. *coli* C600Z1 strains carrying the pZE21-MCS1 vector with a synthesized NDM-1 gene insert or no insert were used as positive and a negative controls, respectively. Additionally, *Klebsiella pneumoniae* CCUG 64452 (OXA-48), *K*. *pneumoniae* CCUG 56233 (KPC-2), and *K*. *pneumoniae* CCUG 58547 (VIM-2) were used as positive controls; and *Enterobacter cloacae* CCUG 59627 (AmpC combined with decreased porin expression), *K*. *pneumoniae* CCUG 59359 (TEM-52), and *E*. *coli* CCUG 17620 (an antibiotic sensitive type strain) were used as negative controls for the Carba NP test.

### Phylogenetic analysis

Phylogenetic analysis was performed to summarize the results and to visualize the evolutionary relationships between the predicted B1BL genes. First, redundant genes were removed by clustering the sequences with a 100% sequence identity cut-off using USEARCH with the parameters “-cluster-fast <retrieved-sequences.fasta> -id 1 –centroids <unique-genes.fasta> -sort length –uc <cluster-file.uc>.” The set of representative genes for each cluster (the centroids) was then spiked with the previously confirmed genes from the B1, B2, and B3 subclasses, respectively. Glyoxalase II (NP_973679.1) from the MBL superfamily was used as an outgroup [58]. The genes were divided into gene families by clustering at a 70% amino sequence identity cut-off using USEARCH with the parameters: “-cluster_fast <fasta-file> -id 0.7 –centroids <centroids.out> -sort length –uc cluster.out.uc.” Clusters that did not contain any previously experimentally confirmed B1BL genes were annotated as novel gene families. A phylogenetic tree was created based on this set of centroid genes using ETE NPR [72] with the parameters “-a < centroid_file > −w standard_fasttree.” The predicted genes and families were all given a consecutive identifier abbreviated as “GXX” and “family Y.” Furthermore, predicted genes that were experimentally confirmed to hydrolyze imipenem were named according to current nomenclature. Finally, the genetic context of the selected genes was annotated using NCBI GenBank, ResFinder, and CONJscan-T4SSscan.

## Additional files

Additional file 1: Figure S1. A flowchart of the method utilized in this study. The left side shows the flow for genomic data, while the right side shows the flow for fragmented metagenomic data. (PDF 30 kb)

Additional file 2: Table S1. A list of the predicted previously uncharacterized B1BL genes together with their amino acid sequences. (XLSX 26 kb)

Additional file 3: Table S2. A list of the subclass B1 metallo-β-lactamases that were included in the development of the hidden Markov model (HMM). (DOCX 12 kb)

Additional file 4: Figure S2. The fraction of identified full-length genes (left) and fragmented sequences (right) as a function of the domain score from HMMsearch. The true positive rate was obtained by a leave-one-out cross-validation while the false positive rate was obtained by feeding the developed HMM both full-length negative sequences and fragmented negative sequences. (PDF 21 kb)

Additional file 5: Table S3. Metagenomic data sets used in this study. (DOCX 12 kb)
