# Supplementary figures and images for: Identification of 76 novel B1 metallo-β-lactamases through large-scale screening of genomic and metagenomic data
Source: Microbiome. 2017 Oct 12;5:134. doi: 10.1186/s40168-017-0353-8 (PMC5637372; doi:10.1186/s40168-017-0353-8)

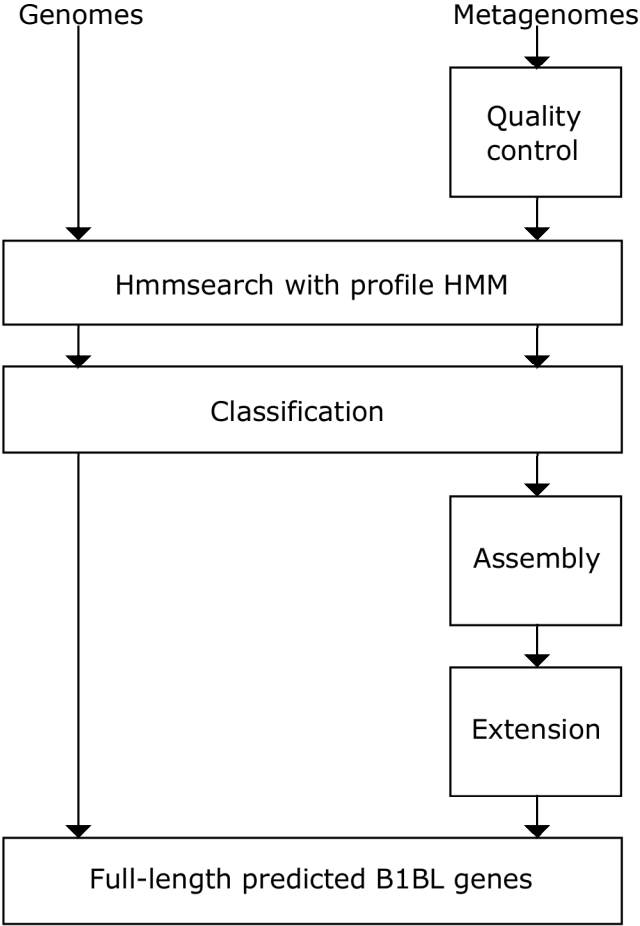

Supplement: Supplementary file 1 — A flowchart of the method utilized in this study. The left side shows the flow for genomic data, while the right side shows the flow for fragmented metagenomic data. (PDF 30 kb) [file 40168_2017_353_MOESM1_ESM.pdf]

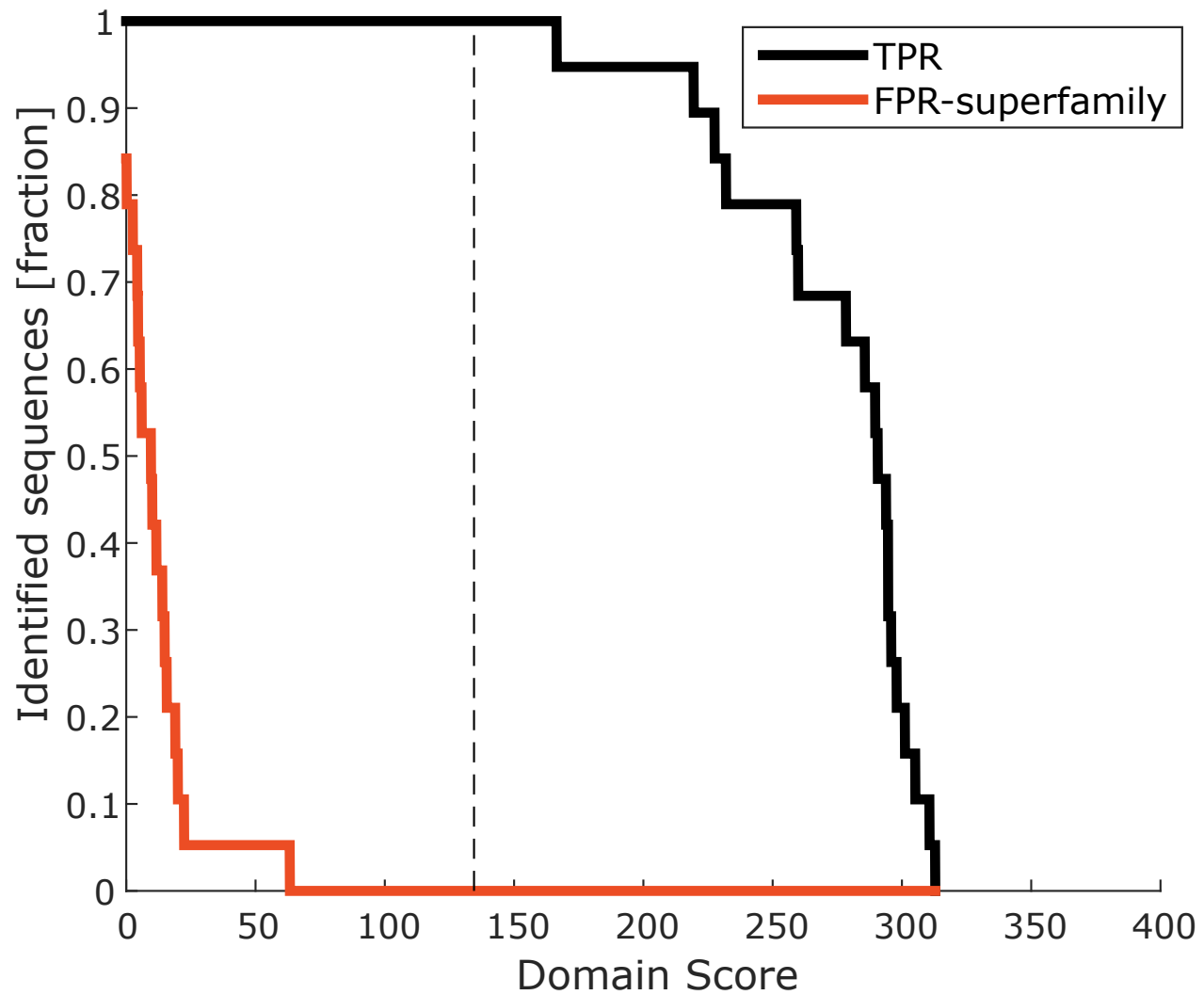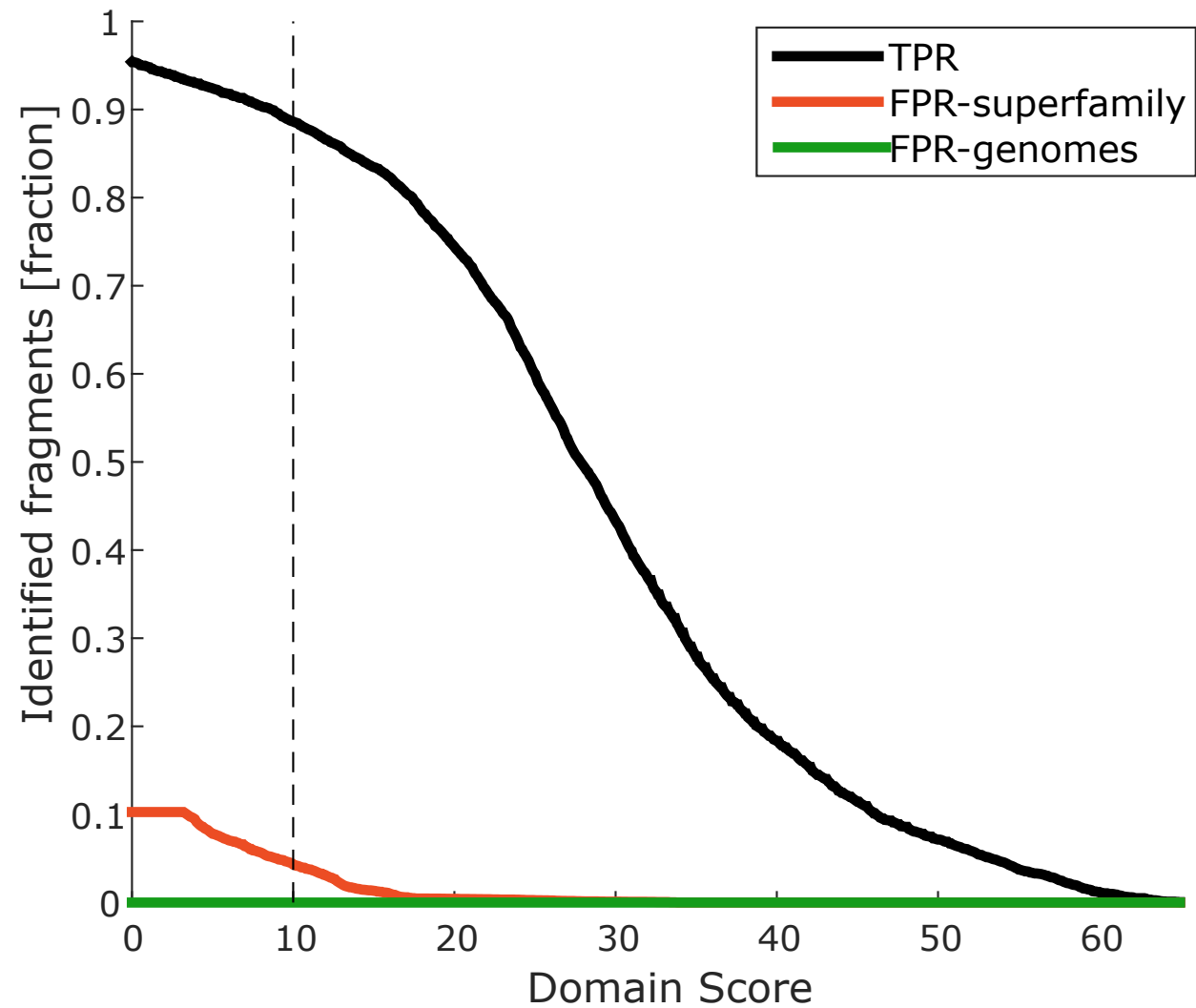

Supplement: Supplementary file 4 — The fraction of identified full-length genes (left) and fragmented sequences (right) as a function of the domain score from HMMsearch. The true positive rate was obtained by a leave-one-out cross-validation while the false positive rate was obtained by feeding the developed HMM both full-length negative sequences and fragmented negative sequences. (PDF 21 kb) [file 40168_2017_353_MOESM4_ESM.pdf]
