# Supplementary material for: Identification of 76 novel B1 metallo-β-lactamases through large-scale screening of genomic and metagenomic data
Source: Microbiome. 2017 Oct 12;5:134. doi: 10.1186/s40168-017-0353-8 (PMC5637372; doi:10.1186/s40168-017-0353-8)
Supplement: Supplementary file 3 — A list of the subclass B1 metallo-β-lactamases that were included in the development of the hidden Markov model (HMM). (DOCX 12 kb) [file 40168_2017_353_MOESM3_ESM.docx]

| **Metallo-β-lactamase**  **Subclass B1** | **Accession**  **no. (Protein)** |
| --- | --- |
| IMP-1 | AAB30289.1 |
| TMB-1 | CBY88906.1 |
| EBR-1 | AF416700_1 |
| GIM-1 | CAF05908.1 |
| KHM-1 | BAH16555.1 |
| DIM-1 | ADD91577.1 |
| CGB-1 | AF339734_1 |
| SPM-1 | CAD37801.1 |
| BcII | AAA22276.1 |
| Bla2 | ACQ49529.1 |
| JOHN-1 | AAK38324.1 |
| NDM-1 | CAZ39946.1 |
| MUS-1 | AAN63647.1 |
| SIM-1 | AAX76774.1 |
| BlaB-1 | AAF89154.1 |
| VIM-1 | CAB46686.1 |
| FIM-1 | AFV91534.1 |
| CcrA | AAA22904.1 |
| TUS-1 | AAN63648.1 |
| IND-1 | ABO21411.1 |
