# Supplementary material for: Identification of 76 novel B1 metallo-β-lactamases through large-scale screening of genomic and metagenomic data
Source: Microbiome. 2017 Oct 12;5:134. doi: 10.1186/s40168-017-0353-8 (PMC5637372; doi:10.1186/s40168-017-0353-8)
Supplement: Supplementary file 5 — Metagenomic data sets used in this study. (DOCX 12 kb) [file 40168_2017_353_MOESM5_ESM.docx]

| **Dataset** | **No. samples** | **Description** | **Reference** |
| --- | --- | --- | --- |
| Isakavagu river | 6 | Pharmaceutical polluted river sediments | [14] |
| Kazipally lake | 1 | Pharmaceutical polluted lake | [12] |
| Patancheru soil | 15 | Soil | [58] |
| Patancheru well | 13 | Well water | [58] |
| WWTP | 70 | Waste water treatments plant | [13] |
| Oil spill | 14 | Oil exposed marine sediments | [57] |
| Pune river | 12 | River sediments | [59] |
| Human gut 1 | 124 | Human gut microbiome | [55] |
| Human gut 2 | 114 | Human gut microbiome | [56] |
| HMP | 764 | Human micriobiome | [49] |
